# A Treatise on Obstructed and Inflamed Hernia

**Published:** 1829-07-01

**Authors:** 


### A TREATISE ON OBSTRUCTED AND INFLAMED HERNIA, AND ON MECHANICAL OBSTRUCTIONS OF THE BOWELS INTERNALLY; AND ALSO AN APPENDIX, CONTAINING A BRIEF STATEMENT OF THE CAUSE OF DIFFERENCE IN SIZE OF THE MALE AND FEMALE BLADDER. By *Henry Stephens*, Member of the Royal College of Surgeons. Octavo, pp. 191. London, E. Cox, 1829.

THE author informs his readers, in a modest and sensible preface, that he does not propose to write a general treatise upon hernia, nor compete with the great names of Pott and Hey, Lawrence and Cooper, that shine so brightly and broadly on this division of surgical science. His object has been rather to fill up lacunæ, to supply some omissions and defects in the works already existing, and to describe some varieties of hernia which he avers to have been yet unnoticed by other authors. We shall presently see how far Mr. Stephens will succeed. The author begins with a chapter on "Obstructed Hernia," but it exhibits a sad want of systematic arrangement, and is put together in so rambling a manner, that any analysis would be quite out of the question. Under these circumstances, we must let Mr. Stephens explain his own views, which he does by a case.

"Long existing and irreducible herniæ produce many remarkable and painful symptoms, and, I believe, frequently occasion death, from a cause which it is in the power of surgery to remove by an operation, where the true nature of the malady is well understood.

"A remarkable case of this kind came under my care, where I had the satisfaction of saving the life of my patient by an operation, although it was one of those cases which have not been considered as requiring it, no strangulation existing.

"In the month of September, 1824, a woman was taken with sickness, and pain in the bowels. Cholera morbus being then prevalent, I expected that these were the preceding symptoms of such an attack; and, as the sickness was not followed by the usual action on the bowels, I gave her some aperient medicines, to favour the intentions of nature. Contrary, however to my expectations, no action of the bowels took place; the pain, though frequent, was not constant, nor yet alarming, nor was the vomiting. I was called to her first on the Thursday, she having then laboured under the above symptoms three days; she continued in the same state for three days longer, without any action of the bowels, notwithstanding my continued endeavours to procure it by aperients and injections. At this period the matter vomited had a fœcal appearance, which the next day was increased, with a very offensive smell. The pain was not severe, and was not felt much, except on turning upon the left side: this position caused pain and vomiting, so also did the taking of either food or medicine; there was no tension, and but a slight soreness of the abdomen. Considering that this was a case of extraordinary anti-peristaltic action, I gave the croton oil, in doses of three and four drops, with a hope of producing a revulse of the peristaltic movements; this, however, only caused increased vomiting, as did all aperient medicines. I enquired if she was the subject of rupture, she answered that she was not, but as I afterwards found, she did not know what I meant. I was not very minute in my enquiries upon this point, because I did not regard the symptoms as indicating any strangulation of the bowels. Upon the fourth day, her countenance began to exhibit signs of sinking, and the pulse was getting feeble and fluttering; these symptoms slowly and gradually, but progressively increased. After she had continued thus until Thursday, being one week from the day I was first called to her, she slightly mentioned to me that she had a swelling on the side of the abdomen, which had existed twenty years. Upon examining, I found a ventral hernia, having upon its surface the appearance of an old cicatrix. It was situated to the left of the umbilicus, and some little distance below it. Upon enquiry I found this hernia had existed ever since the birth of her first child, and that there had been a sore upon the surface of the skin at its first formation, and that no alteration or change in it had occurred since. It was soon evident that this hernia was not strangulated; it was not tense; pressure upon it gave no pain; it receded under the touch, and passed readily into the abdomen, with a slight gurgling noise, but returned when the pressure was removed. The pain, which had all along been in the bowels, was not of that agonized and alarming character which strangulated intestine always produces; and was not present, nor was the vomiting, except upon turning on the left side, or upon taking any thing into the stomach. I therefore concluded, from the hernia being easily returned, that the illness was not depending upon that, and also that an operation would be useless, as no good purpose could be answered by it. She continued sinking, her pulse becoming excessively feeble and tremulous, her voice failed her, and she was unable to speak, except in a low whisper, and with long drawn sighs. On Friday she was worse, the sense of sinking had increased, and as she feebly expressed it, she felt as if her life was going from her; she had occasional faintings, with frequent hiccup, and the stercoraceous vomiting was still more fœtid and discoloured. From her family I learned that she had long been subject to a complaint in her bowels, always feeling pain after her meals, which was considered as colic; she was often obliged to leave her work in consequence of pain after eating. Reflecting, on my return home, upon the history of this case, I concluded that the symptoms, although not those of strangulated hernia, were yet such as would be produced by any permanent and mechanical obstruction in the bowels. I therefore considered that it was not only *possible* but *highly probable,* that the obstruction was in that portion of the bowel which was contained in the hernial tumour. I therefore determined instantly to cut into the hernial swelling, and examine the condition of the parts, and thus see if relief was possible. I returned to my patient, and proposed to her and her friends an operation, as affording her a chance of relief. She, but more especially her friends, refused to permit any such experiment to be made, and urged, among other things, her apparently dying state. I admitted her extreme danger, but contended that there was a chance of saving her, because many of her present symptoms had existed from the first, and that although extremely ill, yet she was not many degrees worse than she had been for some days. I however could not prevail. Friday passed away, and on Saturday morning I found her with little perceptible alteration. I again urged an operation, and declared to her friends that if they prevented it I should consider them as the cause of her death. I found them, although not absolutely submitting, yet inclined to yield to my persuasions. I directly sent an invitation to two of my professional friends, acquainting them that I had an important and novel case, upon which I was going to operate. On Saturday afternoon they came, and saw the case, and heard my explanation upon it, by which they were well satisfied as to the propriety of my attempt, but feared (as I did also) that it was too late. I had by this time overcome the reluctance of the friends, and although there appeared but a very slight chance of success, I had determined upon trying it. I divided the integuments, which were very thin, by a crucial incision, and afterwards a superficial fascia, with some cellular structure; the hernial sac was now exposed, which I opened freely, and found a portion of the small intestine within, which was irreducible, another portion being loose, and readily passing into the abdomen when pressed upon. The small irreducible knuckle of intestine was adhering very firmly to the hernial sac, and in a position which at once accounted for the symptoms I had observed; it was so closely united by adhesions to the hernial sac as to obstruct, to all appearance, its peristaltic action, and prevent the due course of its contents. There was no stricture, for I passed my finger very easily into the abdomen by the side of the intestine, which was somewhat discoloured. I relieved the bowel from its adhesions to the hernial sac, partly by the knife and partly by the finger, with the assistance of my friends, and pushed the intestine into the abdomen, passing my finger in and around the opening on the inside, to be satisfied that there was no further adhesion. I then sewed up the wound. She did not feel that immediate relief from this operation which is experienced in cases of strangulated hernia. The first favourable symptom which was observed was, that upon swallowing some liquid, she had no vomiting after it, a circumstance which had never occurred previous to the operation. I saw her again in two hours, and found that she had passed some fœcal matter from the bowels, for the first time since her illness, and that the hiccup had very much abated; her countenance now wore a less anxious appearance, but her pulse continued very low and fluttering, and she still felt a great sense of sinking, but rather less than usual. She continued in this manner for three days, slowly improving. I gave her aperient medicines and injections, which did not operate very freely. On the third day I gave her a strong dose of aperients, which produced copious discharges; the pulse after this *immediately* began to rise, the sense of sinking almost instantly went off, and she rapidly recovered." 8.

The above case opened to our author's mind new views of the nature of hernia, which views are, that not only strangulated herniæ may be fatal, but that simple adhesion of the hernia to the sac may also produce symptoms, which, though not so quickly, are not less surely fatal than those occasioned by strangulation. The latter cases are much more gradual in their progress than the former, in the one detailed by Mr. S. the symptoms having lasted a week and two days after he was called to attend, besides having existed three days previous to that. In the umbilical and ventral hernia especially, fatal symptoms are far from uncommon. Disorders, says our author, in the intestinal canal are produced by the adhesions of the hernia to the sac, which have the effect of rendering the peristaltic motion and passage of the ingesta through that portion of the tube, confined, difficult, and painful. Thus umbilical and ventral herniæ are well known to occasion complaints of the stomach, and habitual colics, especially after meals. This difficulty in the performance of the important function of the part goes on increasing with the increase of adhesions, till at length it amounts to a total obstruction, producing death, though with symptoms less violent, and considerably more protracted than in strangulated hernia. Sometimes "this state of adhesion terminates in a direct inflammation of the parts."

In the operation for strangulated hernia, Mr. Stephens recommends the surgeon not to return the bowels, "until he is satisfied that they are so freed from their adhesions, that when returned into the abdomen, they will be capable of resuming their functions." In all doubtful cases he would open the sac, examine the state of the parts within, remove the adhesions when that is practicable, and, if possible, return the contents of the hernia into the abdomen. It is well known, and generally admitted, that umbilical herniæ are generally accompanied with colics, diarrhœas, &c. which, Mr. S. conceives, are strong arguments in favour of the truth of what he has advanced; that adhesion is generally the cause of the previous disorders, or of what he should term the premonitory symptoms, and also, in many cases, of the ultimate fatal effects. Mr. Stephens, however, by no means confines the bad consequences of adhesions to umbilical herniæ, but cites various cases that occurred to Mr. Earle, Mr. Lawrence, Mr. South, Mr. Key, and Sir Astley Cooper, to prove that they occur in the other varieties of the disease.

"Upon a careful perusal of the very excellent 'Treatise on Ruptures,' by Mr. Lawrence, I find under the head of 'Chronic or Slow Kind of Strangulation,' an indirect description of the hernia, to which it is my purpose in this work to call the attention of the profession. Mr. Lawrence has very ably described the different progress of the symptoms in this, and in what he calls the 'Acute, or Inflammatory Strangulation.' The latter, he describes, as more frequently occurring in recent hernia, in young persons; the former in large, and old hernia. Mr. Lawrence's description, although clearly agreeing with a state of obstructed hernia, does not exactly correspond with what I have observed. He describes the strangulation in these cases, as being caused by an accumulation of the intestinal contents in the hernia, which he says, is indicated by a slow swelling or enlargement of the parts, and an enlargement of the abdomen, from accumulation above the stricture. 'The indication,' he observes, 'is to unload the intestine.' In the cases which I have related, mere fæcal accumulation was not the cause of the symptoms, for if so, Nature had herself fulfilled the indication, for in the case which terminated fatally, the stercoraceous vomiting had completely emptied the alimentary canal above the stricture, with the exception of some flatus; and in both cases, the intestine in the hernia was empty.

"Mr. Lawrence has, under the head of slow strangulation, described a state of obstructed hernia from fæcal accumulation, and without doubt, such a state often is produced; but I suspect that the cause will be found to be an adhesion of the parts. Where there is no adhesion, I should imagine that the obstruction might generally be removed by the usual means adopted for reducing hernia, for a collection of fæces only, could hardly oppose an inflexible resistance to the return of the parts; particularly in an early stage of the symptoms. Obstructed Hernia admits of relief, or not, in proportion to the degree of permanence in the cause of obstruction.

"With due respect and deference to the opinion of Mr. Lawrence, I consider that this hernia ought not to be described under the head of strangulation, because the symptoms are produced by a different cause, and frequently exist without any stricture whatever; and upon a true understanding of this distinction between Strangulation and Obstruction, the life of the patient often depends; for without it, if a surgeon in operating upon this kind of hernia, should find a slight degree of constriction, he would content himself with dividing the stricture, considering it as the sole cause of the symptoms; he probably would not think of separating the adhesions, unless easily effected. The patient left in this state, (and most authors justify this proceeding,) would die, and upon dissection, the bowels in the abdomen, and the peritoneum, would probably be found more or less inflamed, which in the mind of the surgeon, would sufficiently account for the patient's death, under the name of *Peritoneal Inflammation*, or the continuance of *Enteritis*." 62.

Mixed cases of obstruction and strangulation Mr. Stephens considers as much more frequent than those of simple obstruction. All previously reducible ruptures which have suddenly descended, producing symptoms of ileus, are without doubt caused by a stricture, but all large and irreducible herniæ, which have for some time previously caused pain, particularly after meals, and have produced occasional obstructions in the bowels, are most probably connected with adhesions. The tumour is also generally less tense, and the abdomen not so soon painful in cases of obstruction, as of strangulation; but these distinctions are not immediately necessary, if a surgeon bears in mind the necessity of separating any adhesions which he may find, in cases where he has occasion to operate for hernia, and of not considering the stricture as the only possible cause of the symptoms,

So much for our author's views of what he denominates "obstructed hernia," and we believe that we have given the pith and marrow of them. No doubt can exist, nor indeed has at any time existed, that bowels confined by adhesions in hernial sacs, will not go on with their natural functions so freely as when loose and floating in their native cavity. But the question is, will adhesions, *per se*, occasion a fatal obstruction to the office of the gut? Mr. Stephens says they will; and the issue is with him and his surgical brethren. For our own parts, we doubt whether such be the case, nor can we imagine that a patient will die from this cause alone. The case is very different when the adhesions are so arranged as to act like a stricture in the gut, or when the latter is so placed as by being convoluted in itself, or in any other manner to prevent the egress of matters from its cavity. We also doubt very much the propriety of the practice recommended by Mr. Stephens, of always dividing the adhesions in the operation for strangulated hernia, and particularly of dissecting, as it were, one fold of gut from the other. Who has not seen adherent intestine left in the sac after the operation of dividing the stricture, and that without any bad results whatever? He would be a bold surgeon, we think, who, in operating for old irreducible herniæ, was to make it a rule to return the parts, no matter how firm the adhesions were! Whilst we profess ourselves not to be convinced then by our author's reasoning, we award him gladly the meed of ingenuity, zeal, and industry in collecting the facts with which he has flanked his opinions. We must say too, and we do it sincerely, that Mr. Stephens has at all events placed in a stronger point of view than is usually done, the inconveniences, if not dangers, of adherent hernia.

Our author next proceeds to the consideration of INFLAMED HERNIA, and although we by no means agree in much that he advances, we cannot but admire the dexterity with which he brings forward other peoples' cases to support his views. Having shewn, or attempted to shew, that adhesions of the gut are a source of obstruction to the right performance of its functions, he conceives that they are also a frequent cause of inflammation.

"The contents of a rupture are said sometimes to become inflamed in connexion with an inflammation of the bowels generally, and totally independent of any cause arising from the rupture. That this may sometimes be the case is probable, but I believe such instances are extremely rare. These inflammations, I believe, are almost always generated by the morbid condition of the parts within the rupture, and afterwards becomes quickly communicated to the interior of the abdomen. Large irreducible herniæ, more especially umbilical, are those in which this form of disease mostly occur, which appears to partake more of the character of enteritis, than of ileus. A small portion of confined intestine, however intensely inflamed in itself, does not so necessarily, or so quickly, communicate its disease throughout the abdomen, it being of comparatively local origin; but when the contents of a large hernia become inflamed, as a sequel (I believe) of various chronic confinements, and changes of structure in the parts, the disease from the first will be of a more diffused and general character, and will more extensively and quickly communicate with the interior.

"Although large irreducible ruptures are those in which disease and inflammation, independent of mere mechanical obstruction, are most likely to arise, yet small irreducible ruptures are also very subject to this form of complaint, particularly omental, or those wherein omentum is contained. The omentum is subject, in its unnatural situation, to become thickened and diseased, and to suppurate.* The hernial sac will also often inflame and suppurate. The appendiculæ epiploicæ of the colon will also occasionally undergo some alteration of structure, when confined within a hernial sac. All these various changes and states of disease become a frequent source of inflammation to the contiguous bowels or peritoneum. The inflammation which is thus produced is attended by an obstinate obstruction, and symptoms of general inflammation throughout the abdomen, and is generally fatal in its consequences. When these herniæ are small, the inflammation of the rupture denoted by pain, soreness, and tension of the part, so clearly precedes the inflammation of the abdomen, that the case is usually mistaken for strangulated hernia, and if an operation is performed, the tension which the parts have acquired fills up the opening through which they have descended, and favours the mistaken opinion of the existence of a stricture. When the herniæ are large, the inflammation of the abdomen and of the hernia are very often nearly simultaneous, and if upon operating there is found a palpable absence of stricture, then the hernia is supposed to be merely participating in a general inflammation of the intestines. The want of success attending operations upon large herniæ, particularly umbilical, is attributed to the direct exposure of the peritoneal cavity, by which a dangerous inflammation is excited. I believe that the inflammation which destroys the patient is, in the majority of cases, altogether established before any operation is attempted.

"Cases of unsuccessful operation for hernia are, in my opinion, very frequently cases of the above kind. If omentum has formed a portion of the hernia,† it is generally found upon dissection to be in a state of suppuration, adhesion, or thickening. Considerable inflammation is usually found to have prevailed throughout the abdominal cavity, and herein consists one strong feature of distinction between inflamed and strangulated hernia In inflamed hernia, the peritoneum and intestines are found inflamed *throughout*. Layers of coagulable lymph, and depôts of pus, are interposed between the convolutions of intestine, both *above* and *below* the part forming the hernia. In strangulated hernia, after death, according to Sir A. Cooper, page 29, Part 1st. 'three or four convolutions of intestine are found lying across the abdomen, so enormously distended as to exclude the other viscera from view, and agglutinated slightly together by an effusion of adhesive matter; the track of adhesion is formed by red lines,' &c. and further, 'the portion of intestine *below* the stricture, is, on the contrary, remarkably contracted in diameter, and *free from any appearance of inflammation.*'" 71.

With regard to the diagnosis of inflamed herniæ, our author remarks, that—

"It may be distinguished from a strangulation of such parts, by the more

---

"* In old irreducible hernia the omentum often becomes diseased."—*Sir Astley Cooper, page 27, Part 1st.*

† Sir Astley Cooper says, page 30, Part 2nd, "I have never seen the umbilical hernia in the adult but that it contained omentum."

gradual approach of the symptoms, and by their less degree of violence. From obstructed hernia from adhesion it is to be distinguished by the pain and tenderness of the parts generally preceding the obstruction, and by there being more decided marks of an inflammation existing. Pain, with inflammation throughout the abdomen, is generally soon manifested in inflamed hernia, whereas, in obstructed hernia, it is a late symptom, and in general, scarcely prevails at all. Obstructed hernia *may* possibly be followed quickly by inflammation, and then it would become altogether as a case of inflamed hernia, and require an earlier operation.

"In inflamed hernia, the viscera of the abdomen are very extensively inflamed throughout. In obstructed hernia, very slight traces of inflammation are in general visible after death. Cases of strangulation, are of an intermediate kind; the inflammation being almost wholly confined to the seat of stricture, and the parts above it; the intestines below being in a state of collapse, and uninflamed.

"An empty hernial sac is not unfrequently, by becoming thickened and diseased, a source of inflammation to the bowels and peritoneum; but I have reason to believe that the inflammation so produced is not generally so extensive or so fatal as when intestine is contained within. Coagulable lymph, or pus, forms within the sac. If the former, adhesive inflammation only has prevailed, and the patient will not unfrequently recover. When pus has formed, the case is more dangerous. An operation appears to do good, by giving exit to any pus or fluid which has been secreted." 90.

The chapter on the Treatment of Hernia we must pass over, as it contains scarcely any thing that has not already been advanced by Mr. Stephens in his Observations on Obstructed and Inflamed Herniæ. A suggestion for the radical cure of the disease is too curious to be dealt with in so summary a manner. We find it quite impossible to analyse the work, and contrary to our usual custom we are constrained to indulge in longer extracts than we could wish. The following will describe the operation proposed by our adventurous author.

"A friend of mine, had a favourite and very valuable pointer bitch, the subject of a very unsightly, and enormous hernia, which from its great size and weight, rendered the animal nearly useless, and her owner had considered the propriety of destroying her. I begged to be allowed to try the effects of an operation to return, and retain, the protruded bowels in the abdomen. From the time the hernia had existed, and from its very large size, I had great doubts of success. I began by reducing the condition of the animal, as I foresaw that the less superfluous fat there was upon the omentum, and in the interior of the abdomen, the greater was the chance of success in returning and retaining the parts. When she was sufficiently reduced, I began the operation by feeling for the opening through which the intestines protruded from the abdomen; upon distinctly feeling this, which was in the situation of the inguinal ring, I began the incision directly over it, carrying it about half way down the surface of the tumour, and through the integuments. I then cut through a quantity of fine cellular structure, and opened the hernial sac, and found omentum and intestines within. I began immediately to draw the parts up from the bottom of the tumour, and to push them with my finger through the opening into the abdomen; but I found there was one considerable portion which I could not reduce, owing to its strong adhesions below. Having always been able apparently to return the hernia, I was surprised to find it irreducible, but it seems, it was the omentum and one portion of intestine only, which was returnable, another portion, being firmly connected to the parts out of the abdomen, had never admitted of reduction. I however proceeded by inverting the hernial tumour, by which means I could see the whole irreducible part of the intestine, without the necessity of laying the sac open to the bottom; this discovered to me that the bowel was not simply adhering to the hernial sac, but that its coats were absolutely incorporated with it, having no line of separation. To attempt in this case to dissect the bowel away from the sac, would have been at a very considerable risk of wounding it; but it occurred to me, that I could separate the sac from the integuments, &c. forming the hernial pouch, to which it had become closely joined. In this I succeeded, and returned the intestine and sac into the abdomen, adhering as I found them. The opening from the abdomen was so considerable, that unless my finger was almost constantly there, I could not prevent the parts from again protruding. The difficulty now, was to retain the bowel within the abdomen. A bandage was of no use, and my object was to gain a radical cure by effectually closing the abdominal opening. I succeeded in preventing the parts from protruding, by means of the quilled suture, substituting pieces of wood for quills; these being drawn closely over the opening, prevented any immediate descent of the hernia; but I saw clearly that the purpose of the operation could not in this way be fulfilled, for the abdominal opening could not be closed by means of the integuments, which would necessarily unite anterior to, and not over the ring, and therefore, the intestines might again force their way beneath them into the cellular structure. However, the immediate return of the hernia was prevented by it, but I must confess, I had but few hopes of its ultimate success. I finished, by closing the remaining part of the wound by sutures. The pressure of the quilled suture upon the vessels of the thigh, obstructed the passage of the returning blood, and caused œdematous swelling to some extent in one limb; I relieved this by incisions, and at the end of about four or five days, removed the sticks and ligatures. The removal of the sutures relieved the swelling, and the animal recovered rapidly. Some physic which I gave her, operated freely, without occasioning any disposition in the parts to return. The operation was performed in August, and the bitch was used during the shooting season of September and October, and proved equal to any exertion that was required. Having subsequently removed the pouch which contained the protruded bowels, no trace of the deformity remained. I had the satisfaction of seeing my canine patient perform her duties with alacrity and vigour; and of receiving with the apparent gratitude of the animal the warmest thanks of her master.

"The radical cure in this case, appeared to me to be owing to the return of the hernial sac. It was separated from a very close adhesion to the hernial pouch, by means of the fingers and the knife, and it was returned in this state of recent separation, into the abdomen, ready to attach and unite itself to any surface to which it was opposed. By its inclination to descend again, it was, although kept in the abdomen, closely applied to the abdominal ring, over the interior of which it without doubt closely adhered, and by such means completed, although in a manner I did not contemplate, the radical cure." 115.

Whether any operation of this kind is likely to be generally adopted by the profession we shall not pretend to decide, suffice it that the above was its issue in Mr. Stephens's canine friend. We fear that our limits will prevent our noticing that portion of the work which treats of mechanical obstruction of the bowels within the abdomen. We may say that the author displays much acuteness in his observations, and great boldness (shall we say temerity?) in his methodus medendi, or rather operandi. Those who wish to learn his particular views may consult the work itself, of which we can sincerely pronounce a favourable opinion, although we are far from agreeing in every respect with the author. It is for the public to decide who is right and who is wrong, and no doubt Mr. Stephens is perfectly willing to submit to its ordeal.

